# AMP-activated protein kinase is required for the anti-adipogenic effects of alpha-linolenic acid

**DOI:** 10.1186/s12986-015-0006-5

**Published:** 2015-03-08

**Authors:** Xihong Zhou, Weiche Wu, Jingqing Chen, Xinxia Wang, Yizhen Wang

**Affiliations:** Key Laboratory of Animal Nutrition and Feed Science, Ministry of Agriculture, Zhejiang Provincial Laboratory of Feed and Animal Nutrition, Institute of Feed Science, Zhejiang University, Hangzhou, Zhejiang 310058 PR China

**Keywords:** Alpha-linolenic acid, AMP-activated protein kinase, β-oxidation, Mitochondrial biogenesis, Adipose tissue

## Abstract

**Background:**

n-3 long chain polyunsaturated fatty acid (n-3 LC PUFA) increases β-oxidation and limits lipid accumulation in adipocytes. The current study was conducted to determine whether their precursor alpha-linolenic acid (ALA) could also exert the above effects and how AMP-activated protein kinase (AMPK) was involved.

**Methods:**

AMPKα1^−/−^, AMPKα2^−/−^ mice and wild-type (WT) mice were fed a high-fat diet (HFD) or HFD with ALA. Body weight was recorded weekly and serum was collected. Adipocytes size and expression of key players involved in mitochondrial biogenesis and lipid oxidation were also measured.

**Results:**

Our results showed an elevated serum adiponectin level and a decreased leptin and insulin level in WT mice fed HFD with ALA when compared with WT mice fed HFD. In addition, dietary ALA decreased epididymal adiposity and adipocytes size in WT mice. At protein level, mitochondrial genes (peroxisome proliferator-activated receptor gamma coactivator 1 alpha [PGC1α] and nuclear respiratory factor-1 [nrf1]) and β-oxidation related genes (carnitine palmitoyltransferase 1A [CPT1a] and peroxisome proliferator-activated receptor alpha [PPARα]) were upregulated by dietary ALA in epididymal fat of WT mice. Consistently, dietary ALA also increased mitochondrial genomic DNA copy numbers. Moreover, lipogenesis was repressed by dietary ALA, indicated by that expression of fatty acid synthase (FAS), acetyl CoA carboxylase (ACC) and stearoyl-CoA desaturase 1 (SCD1) were decreased. However, these aforementioned effects were abolished in the AMPKα1 and AMPKα2 knockout mice.

**Conclusions:**

Our results suggest that ALA could improve adipose tissue function and its anti-adipogenic effects are dependent on AMPK.

## Introduction

Many studies have demonstrated that n-3 long chain polyunsaturated fatty acid (n-3 LC PUFA) could reduce adiposity by improving the dysfunctional lipid metabolism induced by HFD, such as stimulation of lipolysis and inhibition of lipogenesis in liver [1,2], as well as stimulation of fatty acid oxidation in muscle [3]. Moreover, n-3 PUFA could also decrease lipid accumulation in adipocytes [4,5]. And n-3 PUFA exerts these effects not only by upregulating mitochondrial biogenesis and increasing β-oxidation [6], but also by reducing cellularity of white adipose tissue [7]. Although most of the studies declare that eicosapentaenoic (EPA) and docosahexaenoic (DHA) acids exert more pronounced effects on the reduction of adipose tissue mass compared with their precursor alpha-linolenic acid (ALA) [6-8], other studies suggest that ALA could also increase fatty acid oxidation both in vivo [9,10] and ex vivo [11]. Importantly, ALA without converting to DHA and EPA, could reduce lipid accumulation [12].

Given that ALA supplementation could increase ALA content in adipose tissue [9] and the critical role of n-3 PUFA in ameliorating adipose tissue dysfunction, it is demanding to understand how n-3 PUFA modulates fatty acid metabolism in adipose tissue. It is widely accepted that the effects of n-3 PUFA are mainly mediated by peroxisome proliferator activated receptors (PPARs) especially PPARα, since n-3 PUFA are ligands for PPARs [13]. However, other studies report that long chain fatty acids could also regulate carnitine palmitoyltranferase I (CPT1a), which is the rate-limiting enzyme in mitochondrial fatty acid oxidation in a PPARα-independent manner [14,15]. In addition, n-3 PUFA also has many effects including stimulation of 5′AMP-activated protein kinase (AMPK) in adipose tissue [16]. AMPK is a heterotrimeric enzyme that plays key roles in energy homeostasis of adipose tissues. For decades, the role of AMPK in the regulation of white adipose tissue metabolism in terms of triglyceride (TG) storage and release, mitochondrial biogenesis and oxidative capacity has been studied [17]. AMPK could directly interact and phosphorylate PGC-1α [18], which stimulates the transcriptional program of mitochondrial biogenesis and oxidative metabolism [19]. It is also well known that AMPK regulates lipogenesis mainly by phosphorylating acetyl CoA carboxylase (ACC), as well as regulates fatty acid oxidation through CPT1 [20]. Considering the potential roles of AMPK regulatory axis involved in induction of the metabolic switch in adipocytes by n-3 PUFA, the current study was conducted using AMPKα1 or AMPKα2 knockout mice to determine whether catalytic subunit (α1 and α2) of AMPK plays a vital role in these effects.

## Materials and methods

### Animals and diets

AMPKα1 and AMPKα2 knockout mice which were both generated from C57BL/6 mice were originally purchased from the Jackson laboratory. The C57BL/6 mice were used as control. All animals were maintained with unrestricted access to water and food under controlled temperature (22 ± 1°C), humidity and air flow conditions, with a fixed 12-h light–dark cycle (light on from 0800 h to 2000 h). Mice deficient in the catalytic subunit of AMPKα1 or α2 and wild type mice (nine weeks old with an average weight of 26.3 g) were fed on either a 45% high-fat diet (HFD) or a HFD containing 10% ALA (purchased from Aladdin, Ltd (Shanghai, China)), maintaining the total amount of fat at 45%. The HFD are consisted of 45% (kcal%) fat (lard and soybean oil), 20% protein (casein and L-cystine) and 35% carbohydrate (corn starch, maltodextrin, sucrose and vitamin). During the experiment (12 weeks), body weight was recorded weekly and food intake was measured every 3 days. At the end of the experiment, blood was taken from the retro-orbital sinus after 4 h fasting and after cervical dislocation, epididymal and inguinal fat pad were separated and weighted, and then were either immediately fixed in formaldehyde solution for morphology observation or snap-frozen in liquid nitrogen. The present study was approved by the Committee of Experimental Animal Care, Zhejiang University (Hangzhou, China) (Project No. 2012CB124705). All animal care and experimental procedures were under the supervision of the Committee of Experimental Animal Care.

### Plasma biochemical assays

The serum biochemical assays were performed with commercially available kits: adiponectin, leptin and insulin (Cusabio Biotech Co., Ltd).

### Measurement of AMPK activity

AMPK activity was assayed using a CycLex AMPK Kinase Assay kit (Cyclex, Japan, Cat#CY-1182). After adipose tissue was homogenized, the resulted supernatant was collected and the relative AMPK activity was determined according to the manufacturer’s instruction.

### DNA content in adipose tissue and Mitochondrial (mt) DNA analysis

Adipose tissue was weighed and then was digested with proteinase K, and DNA was extracted with phenol:chloroform. DNA content was measured by spectrophotometry (λ = 260 nm). mtDNA content relative to nuclear DNA (nDNA) content was assess by qPCR, using 2 ng of total DNA as template and primers for cytochrome c oxidase 2 (COX2, mitochondrial genome; forward: ataaccgagtcgttctgccaat; reverse: tttcagagcattggccatagaa) and ribosomal protein s18 (rps18, nuclear genome; forward: tgtgttaggggactggtggaca; reverse: catcacccacttacccccaaaa) were used.

### Histology and cell-size measurement

Tissue samples of epididymal and inguinal white adipose tissue were fixed with 4% paraformaldehyde and paraffin embedded. Sections of 8 μm were stained with hematoxilin-eosin (HE) and then mean adipocyte surface area was analyzed. Six different representative microscopic fields were captured manually from sections of each sample (three samples per group) and cell size was analyzed using Image-pro plus 6.0 software. The mean adipocyte area was calculated from over 100 cells per animals in each group.

### Immunoblotting

Adipose tissue was homogenised and lysed on ice for 30 min in lysis buffer with 1‰ DTT, 5‰ PMSF and 1‰ protease inhibitor (KeyGEN BioTECH, Nanjing, China). The tissue extract was cleared from fat, nuclei and debris by centrifugation at 13,000 × g for 10 min. Protein content was determined by BCA assay and identical amounts of proteins (20 μg/lane) were separated by SDS-PAGE and blotted onto nitrocellulose membranes. The membranes were blocked with skim milk. Then primary antibody against AMPKα1, phospho-AMPKα2, FAS, ACC, PPARα, PGC1α (Abcam), phospho-AMPKα1, nuclear respiratory factor-1 (nrf1), stearoyl-CoA desaturase 1 (SCD1), CPT1a (Santa Cruz), uncoupling protein 2 (UCP2), AMPKα2 (GeneTex), Glut4 (SAB) and GAPDH (Boster, Wuhan, China) were applied overnight at 4°C. After incubating with the secondary antibody for 1 h at room temperature, the membrane was detected using the EZ-ECL (Biological Industries).

### Statistical analysis

All data were expressed as mean ± SE. Data were analyzed by a one-way analysis of variance (ANOVA) with Tukey’s post hoc test using SPSS (version 16.0; SPSS, Inc.) and significance was accepted at P < 0.05.

## Results

### Effects of ALA on serum insulin, adiponectin and leptin concentration in WT, AMPKα1^−/−^ and AMPKα2^−/−^ mice

As shown in Table 1, dietary ALA significantly decreased serum insulin and leptin concentration of WT mice, while increased serum adiponectin concentration. However, such changes were not observed in *AMPKα1*^*−/−*^ and *AMPKα2*^*−/−*^ mice when fed with ALA.

**Table 1:** **Serum biochemical assays**

	**WT**		**AMPKα1** ^**−/−**^		**AMPKα2** ^**−/−**^	
	**HF**	**HF-A**	**HF**	**HF-A**	**HF**	**HF-A**
Insulin (ng/mL)	3.23 ± 0.39	1.43 ± 0.13^ab^	2.87 ± 0.41	2.42 ± 0.28	2.64 ± 0.33	2.55 ± 0.35
Adiponectin (μg/mL)	4.43 ± 0.83	9.73 ± 2.16^ab^	5.18 ± 1.09	6.29 ± 1.32	5.31 ± 1.53	6.82 ± 2.01
Leptin (ng/mL)	37.5 ± 4.1	22.1 ± 3.7^ab^	39.9 ± 5.3	36.4 ± 4.1	38.2 ± 6.2	34.1 ± 3.4

Note: The values are means ± SE. ^a^P < 0.05 for difference between different genotypes with HF-A diet; ^b^P < 0.05 for difference between WT mice. HF, mice fed high-fat diet; HF-A, mice fed high-fat diet with ALA.

### Effects of ALA on HFD-induced fat deposition in white adipose tissue required AMPK

**Figure 1:**
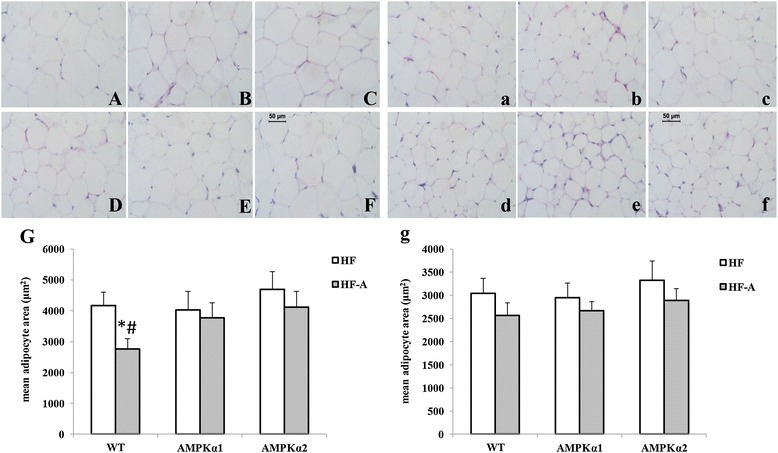
**Adipocyte size in WT, AMPKα1** ^**−/−**^ **and AMPKα2** ^**−/−**^ **mice. A**, **a**, WT mice fed HF diet; **B**, **b**, AMPKα1^−/−^ mice fed HF diet; **C**, **c**, AMPKα2^−/−^ mice fed HF diet; **D**, **d**, WT mice fed HF-A diet; **E**, **e**, AMPKα1^−/−^ mice fed HF-A diet; **F**, **f**, AMPKα2^−/−^ mice fed HF-A diet; **G**, **g**, mean adipocyte area in WT mice, AMPKα1^−/−^ and AMPKα2^−/−^ mice (uppercase represents epididymal fat; lowercase represents inguinal fat; cell size, μm^2^); HF, mice fed high-fat diet; HF-A, mice fed high-fat diet with ALA.

As shown in Table 2, ALA supplementation significantly decreased end point body weight in WT, while ALA supplementation did not significantly affect body weight gain in *AMPKα1*^*−/−*^ and *AMPKα2*^*−/−*^ mice. Dietary ALA significantly decreased epididymal fat accumulation and increased DNA copies in WT mice with HFD, while such effects were not observed in *AMPKα1*^*−/−*^ and *AMPKα2*^*−/−*^ mice. Although inguinal fat content tended to be decreased by ALA in all genotypes, such changes did not show statistical significance. Our results also showed that cell size of both epididymal and inguinal adipocytes in mice of all genotypes tended to be decreased after ALA supplementation (Figure 1). However, only the decrease of cell size in WT mice supplemented with ALA reached significance (Figure 1G).

**Table 2:** **Effects of ALA on fat depots of WT, AMPKα1** ^**−/−**^ **and AMPKα2** ^**−/−**^ **mice fed HFD**

	**WT**		**AMPKα1** ^**−/−**^		**AMPKα2** ^**−/−**^	
	**HF**	**HF-A**	**HF**	**HF-A**	**HF**	**HF-A**
Final BW (g)	42.6 ± 3.1	32.6 ± 1.8^ab^	43.1 ± 3.0	42 ± 3.2	45.5 ± 3.4	44.2 ± 3.7
Epididymal						
weight (mg)	2270 ± 158	1224 ± 98^ab^	2242 ± 201	2137 ± 198	2349 ± 178	2146 ± 184
DNA (μg/mg)	0.32 ± 0.04	0.67 ± 0.05^ab^	0.29 ± 0.05	0.35 ± 0.06	0.29 ± 0.05	0.36 ± 0.07
Inguinal						
Weight (mg)	1090 ± 42	1037 ± 57	1097 ± 45	1058 ± 55	1102 ± 49	1072 ± 59
DNA (μg/mg)	0.42 ± 0.05	0.5 ± 0.06	0.36 ± 0.04	0.39 ± 0.06	0.38 ± 0.05	0.41 ± 0.07

Note: The values are means ± SE. ^a^P < 0.05 for difference between different genotypes with HF-A diet; ^b^P < 0.05 for difference between WT mice. BW, body weight. HF, mice fed high-fat diet; HF-A, mice fed high-fat diet with ALA.

### Effects of dietary ALA on mitochondrial biogenesis and fatty acid oxidation in white fat required AMPK

**Figure 2:**
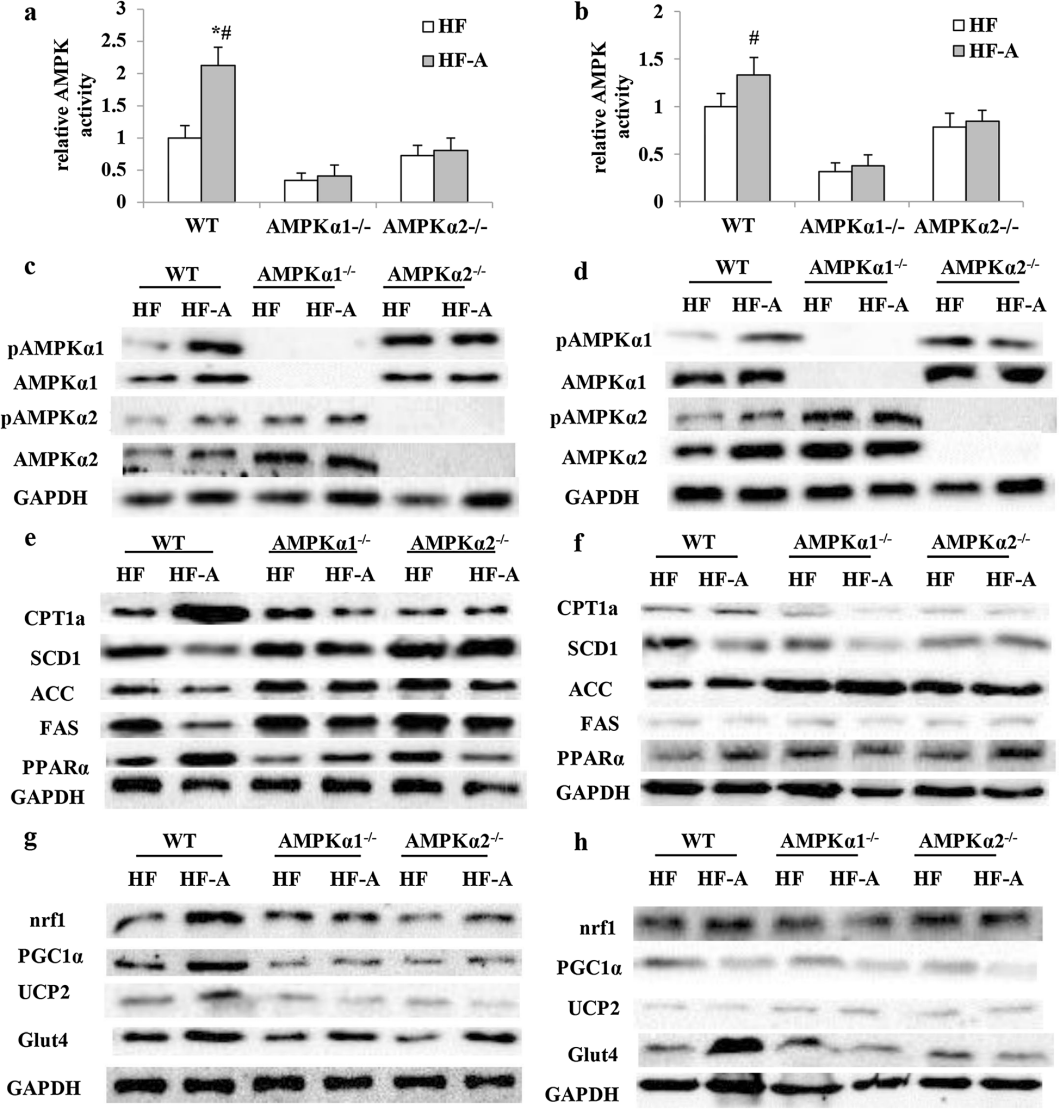
**AMPK activity, protein expression of mitochondrial biogenesis and β-oxidation related genes in WT, AMPKα1** ^**−/−**^ **and AMPKα2** ^**−/−**^ **mice.** HF, mice fed high-fat diet; HF-A, mice fed high-fat diet with ALA. (**a**, **c**, **e**, **g**, activity of AMPK and protein expressed in epididymal fat; **b**, **d**, **f**, **h**, activity of AMPK and protein expressed in inguinal fat). The values are means ± SE. #P < 0.05 for difference between different genotypes with HF-A diet; *P < 0.05 for difference between WT mice.

Compared with WT fed with HFD, AMPK activity in epididymal fat in WT mice supplemented with ALA was significantly higher (Figure 2a). Moreover, AMPK activity in both epididymal and inguinal fat in WT mice supplemented with ALA were significantly higher than that in either *AMPKα1*^*−/−*^ or *AMPKα2*^*−/−*^ mice supplemented with ALA (Figure 2a, b). Compared with WT, protein expression of AMPKα2 were higher in HFD fed *AMPKα1*^*−/−*^ mice and protein expression of AMPKα1 were higher in HFD fed *AMPKα2*^*−/−*^ mice (Figure 2c, d). In WT mice, ALA promoted protein level of both AMPKα1 and AMPKα2 in epididymal and inguinal fat tissues, while such effects were not observed in either *AMPKα1*^*−/−*^ or *AMPKα2*^*−/−*^ mice. Moreover, phosphorylation of both AMPKα1 and AMPKα2 in epididymal and inguinal fat were higher in HF-A group than that in HF group (Figure 2c, d).

**Figure 3:**
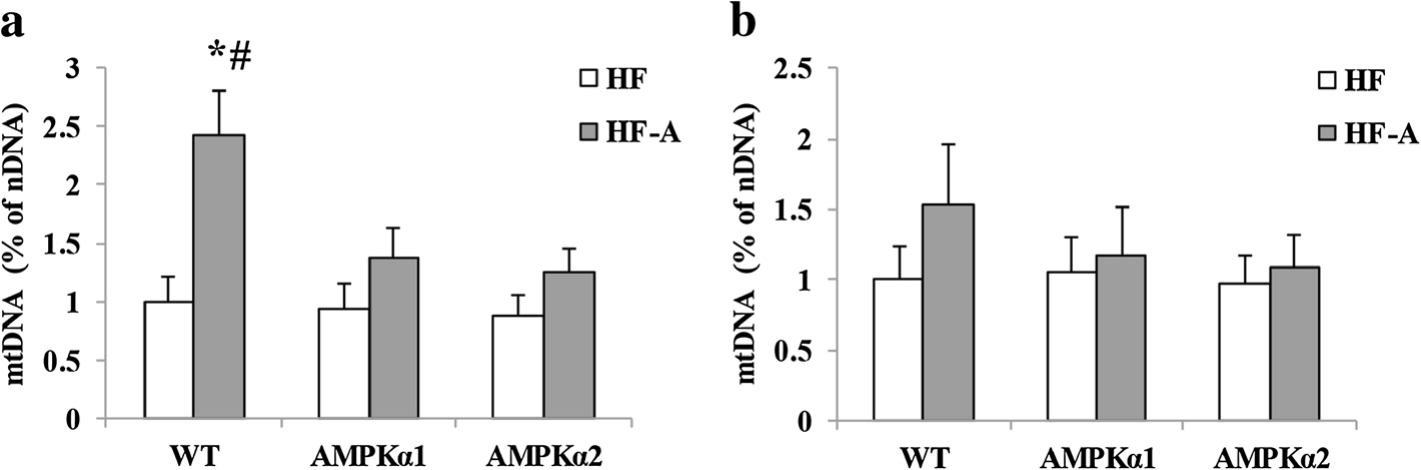
**Relative mitochondrial DNA content assessment.** HF, mice fed high-fat diet; HF-A, mice fed high-fat diet with ALA. (**a**. epididymal fat; **b**, inguinal fat). mtDNA, mitochondrial DNA; nDNA, nuclear DNA; % of nDNA, mtDNA content relative to nDNA content. The values are means ± SE. #P < 0.05 for difference between different genotypes with HF-A diet; *P < 0.05 for difference between WT mice.

Dietary ALA increased protein expression of CPT1a and PPARα, and decreased protein expression of SCD1, ACC and FAS in epididymal fat of WT mice, while not in those of *AMPKα1*^*−/−*^ or *AMPKα2*^*−/−*^ mice (Figure 2e). Moreover, neither ALA supplementation nor genotype remarkably affected protein expression of CPT1a, PPARα, SCD1, ACC and FAS in inguinal fat (Figure 2f). ALA supplementation increased protein expression of PGC1-α, nrf1 and UCP2 in epididymal fat of WT mice, but not in those of *AMPKα1*^*−/−*^ or *AMPKα2*^*−/−*^ mice (Figure 2g). Neither ALA supplementation nor genotype remarkably affected protein expression of PGC1α, nrf1 and UCP2 in inguinal fat (Figure 2h). However, dietary ALA increased membrane Glut4 expression in both epididymal and inguinal adipocytes of WT mice (Figure 2g, h). In accordance with the changes of PGC1-α expression, ALA treatment increased mtDNA copy numbers in epididymal fat of WT mice while not in *AMPKα1*^*−/−*^ or *AMPKα2*^*−/−*^ mice (Figure 3a). However, neither genotype nor diet significantly affected mtDNA copy numbers in inguinal fat (Figure 3b).

## Discussion

Adipose tissues play independent roles in energy homeostasis. The development of metabolic syndrome in obesity was often accompanied with impaired adipose tissue glucose and lipid metabolism. Specifically, adipose tissue is an important target of n-3 PUFA’s effect on metabolic syndrome and adiposity [16]. Previous studies found that EPA and DHA could not only upregulate mitochondrial biogenesis and increase β-oxidation in white fat [6], but also limit hyperplasia and hypertrophy of adipocytes [7]. Consistently, we found that ALA, the precursors of n-3 LC PUFA, could also reduce the accumulation of fat mass induced by HFD mainly through increasing the content of PGC1α, nrf1, UCP2 and CPT1a in epididymal fat, but less effects was observed in inguinal white fat. The site-specific effects of n-3 PUFA on adipose tissue has been demonstrated for decades [4,21,22] as it is widely accepted that the unfavorable metabolic effects of adiposity were particularly resulted from the expanded visceral fat depot [23]. However, the hypertrophy of both subcutaneous and visceral fat depots was both limited after feeding rats for 6 months [24]. As a result, the effects of n-3 PUFA on adipose tissue may also dependent on the duration of dietary treatment.

AMPK is involved in the regulation of white adipose tissue metabolism [17] and is also one of the important mechanisms that mediate the effects of n-3 PUFA on lipid catabolism [16]. Our results showed that the beneficial effects of ALA on lipid metabolism in epididymal fat were diminished in the absence of either AMPKα1 or AMPKα2. These results further confirmed that AMPK was crucial for the effects of n-3 PUFA on adipocytes. Previous studies suggest that the α1 catalytic subunit of AMPK accounts for most of the activity of this kinase in white adipose tissue [25,26]. However, loss of AMPKα1 do not affect the phosphorylation of ACC and HSL in white adipose tissue [27]. Interestingly, lipid deposition in white adipose tissue induced by HFD is increased in AMPKα2 knockout mice. Accordingly, we speculated that both α1 and α2 subunit of AMPK were critical for the effects of n-3 PUFA on adipocytes [28].

n-3 PUFA was found to increase PPARα activity as they are intrinsic ligands for PPARs [29-31] and activation of PPARα promotes mitochondrial lipid oxidation [32]. We found that PPARα expression was increased in epididymal fat by ALA treatment of WT mice instead of AMPKα1 or AMPKα2 knockout mice. Since previous studies demonstrate that AMPK activation enhances the activity of PPARα [33,34], AMPK could be an upstream factor which affects the interaction of n-3 PUFA and PPARα. PGC-1α is a master regulator of mitochondrial biogenesis and stimulates oxidative metabolism [19,35]. Moreover, AMPK initiates many of the important gene regulator functions through phosphorylation of PGC-1α [18,36]. Our results showed that the effects of ALA on the increasing expression of PGC-1α and other related factors such as nrf1 and UCP2 in the epididymal fat of HFD treated mice were diminished when either AMPKα1 or AMPKα2 were deleted. These results further confirmed that AMPK were crucial for the effects of ALA on mitochondrial biogenesis. Moreover, the increased mtDNA copy number after ALA treatment in epididymal adipocytes of WT mice suggested that mitochondrial function was also improved. CPT1a controls the rate of β-oxidation in white adipose tissue [37] while ACC and FAS are key factors for fatty acid synthesis. And these factors are all downstream targets of AMPK [38]. Our results found that expression of CPT1a was increased while expressions of ACC and FAS in epididymal fat were decreased after ALA treatment in WT mice. However, we did not observe any changes in these protein expressions in AMPKα1 or AMPKα2 knockout mice treated with ALA. These results suggested that both AMPKα1 and AMPKα2 were required for the effects of ALA on fatty acid metabolism. Moreover, ALA supplementation increased circulating adiponectin levels, and decreased insulin and leptin levels, whereas these three hormones were not changed in AMPKα1 or AMPKα2 knockout mice, which further implied that ALA had the potential to improve adipose tissue function and AMPK was indispensable for these effects of ALA.

In obesity, Glut4 expression is decreased in adipose tissue and adipose-selective deletion of the Glut4 gene impairs insulin action in muscle and liver [39]. Our results showed that ALA treatment significantly increased membrane Glut4 expression in epididymal fat of mice with HFD, while these effects were diminished when loss of either AMPKα1 or AMPKα2. AMPK activation causes Glut4 translocation in skeletal muscle [40]. However, although studies address that AMPK increases glucose uptake in adipose cells [41], the related mechanism has not been declared. Our results could evidence that AMPK may mediate the effects of n-3 PUFA through inducing the translocation of Glut4 to the membranes of adipocytes.

## Conclusions

In conclusion, our results demonstrated that ALA could mediate its beneficial effects on HFD-induced lipid accumulation mainly by preventing adipocytes hypertrophy and induction of mitochondrial biogenesis and β-oxidation. AMPK is indispensable for the beneficial effects of ALA on adipose tissue metabolism, especially on fatty acid oxidation. These findings might shed lights to the mechanism of the protective effect of n-3 PUFA against obesity.

## Abbreviations

LC PUFA: Long Chain Polyunsaturated Fatty Acid
AMPK: AMP-activated protein kinase
ALA: Alpha-linolenic acid
WT: Wild-type
HFD: High-fat diet
PGC1α: Peroxisome proliferator-activated receptor gamma coactivator 1 alpha
nrf1: Nuclear respiratory factor-1
CPT1a: Carnitine palmitoyltransferase 1A
PPARα: Peroxisome proliferator-activated receptor alpha
FAS: Fatty acid synthase
ACC: Acetyl CoA carboxylase
SCD1: Stearoyl-CoA desaturase 1
EPA: Eicosapentaenoic
DHA: Docosahexaenoic
TG: Triglyceride
mtDNA: Mitochondrial DNA
nDNA: Nuclear DNA
COX2: Cytochrome c oxidase 2
rps18: Ribosomal protein s18
UCP2: Uncoupling protein 2
